# Modified buried vertical mattress suture technique for excisional suturing of benign parotid tumors: A retrospective study

**DOI:** 10.1111/jocd.16521

**Published:** 2024-08-19

**Authors:** Xiangyu Liu, Xueyuan Yu, Yuan Guo, Maoguo Shu, Ming Zhang

**Affiliations:** ^1^ Department of Aesthetic Plastic and Craniofacial Surgery The First Affiliated Hospital of Xi'an Jiaotong University Xi'an Shaanxi China; ^2^ Department of Radiology The First Affiliated Hospital of Xi'an Jiaotong University Xi'an Shaanxi China

**Keywords:** benign parotid tumor, buried suture technique, pleomorphic adenoma, scar

## Abstract

**Aim:**

The purpose of this retrospective patient chart review was to analyze the clinical data of 52 patients with benign parotid tumors who underwent modified buried vertical mattress sutures and to assess the postoperative complication rate and patient scarring.

**Methods:**

A total of 52 patients with benign parotid tumors underwent total parotidectomy and modified buried vertical mattress suture. Variables included general characteristics (age, gender, tumor diameter, and pathologic type), surgical indicators (suture time, wound healing time, operative time, hospital stay, bleeding volume, and drainage volume), complication rates, and Sunnybrook facial neurological function score, visual scar scale (VSS) score and patient and observer scar assessment scale (POSAS) score.

**Results:**

Most tumors were less than 3 cm in diameter, with pleomorphic adenomas being the most common. Suture time was 14.83 ± 1.61 min, operative time was 58.90 ± 15.76 min and hospital stay were 5.12 ± 0.96 days. Postoperatively, salivary fistulae developed in one patient, Frey's syndrome in two patients, temporary facial paralysis in six patients and temporary numbness in the incision area in six patients. At 6 months postoperatively, 86.5% of patients had a Sunnybrook score of more than 80, and VSS scores and POSAS scores were between one and two.

**Conclusion:**

The postoperative complication rate was 30.8%, and the scarring in the facial incision area was mild and close to normal skin at 3 years postoperatively.

## 1 INTRODUCTION

Salivary gland tumors include minor and major salivary gland tumors located in the parotid, submandibular, and sublingual glands. Parotid gland tumors are more common among salivary gland tumors and are predominantly benign. Benign parotid tumors include pleomorphic adenomas, Warthin's tumors, eosinophilic cell tumors, basal cell adenomas, myoepitheliomas, and hemangiomas. Of these, pleomorphic adenomas and Warthin's tumors account for 93% of all benign parotid tumors.^1^ Usually, benign parotid tumors are characterized by a relatively slow growth rate, clear boundaries, a complete envelope, no spread or metastasis, and generally no recurrence through surgical removal. If the patient presents with pain and a rapidly growing mass, suspicion of malignancy should be increased. Total parotidectomy, lateral or superficial parotidectomy, partial lateral parotidectomy, and extracapsular dissection of the parotid gland are currently being selected for the treatment of benign parotid tumors.^2^ The goal of these procedures is to completely remove the lesion and preserve the facial nerve. With the development of society and the economy, patients, especially young women, with benign parotid tumors are not only concerned about the quality of life after surgery, but also have a special concern for facial aesthetics.^3^

Patients with benign parotid tumors place higher demands on surgical suturing techniques due to poor incision healing and scar proliferation leading to poor cosmetic status. The tension‐reducing effect disappears after the removal of the traditional interrupted sutures, which are prone to scarring and poor healing of the incision.^4^ Modified buried vertical mattress suture, a new surgical skin suturing technique, allows the suture to be buried under the epidermis, which helps to improve aesthetics by minimizing skin scarring in patients after surgery. This technique has been used in other clinical departments in recent years and has achieved remarkable results.^5^ In this study, patients with benign parotid tumors who were to undergo parotidectomy were selected to observe the effect of the modified buried vertical mattress suture technique in incision closure.

## 2 METHODS

This was a retrospective patient chart review employing a consecutive sampling method. We reviewed cases of patients with benign parotid tumors operated from 2020 to 2023. Inclusion criteria: (1) benign tumor of parotid gland diagnosed by imaging and pathology; (2) age 18–75 years; (3) underwent total parotidectomy and modified buried vertical mattress suture. Exclusion criteria: (1) suspected malignant lesions of parotid tumors; (2) the presence of severe cardiac, hepatic, pulmonary, renal, and other organ diseases; (3) the presence of contraindications to surgery such as coagulation disorders or diseases of the immune system; (4) undergoing other treatments or trials that may affect the results assessed in this study. Finally, a total of 52 patients were enrolled in this study. This study was approved by the ethics committee. For research in humans, the research was conducted per the principles of the Declaration of Helsinki. To better visualize the surgical results and patient recovery, we provide an image of the appearance of the parotid surgery area in one patient in Figure 2. This patient has signed a written informed consent for the use of anonymized images for publication.

### 2.1 Clinical data

There were 52 cases in this group. General characteristics included the patient's gender, age, tumor size, tumor position, and pathology type. In addition, surgical suture time, wound healing time, operative time, hospitalization time, bleeding, three‐day drainage, and the occurrence of complications were observed and recorded. Facial nerve function was assessed 6 months postoperatively by the Sunnybrook Facial Nerve Rating System. Scarring of the surgical area was assessed at 6 months postoperatively by the Visual Scar Scale (VSS) and Patient and Observer Scar Assessment Scale (POSAS).

### 2.2 Preoperative examination

Before admission, a routine physical examination was performed. The mass was palpably mobile with a clear boundary and moderate texture. The patient was asked for their history and subjective feelings, if they had any pain or discomfort and if the mass was slow growing, and an ultrasound examination was performed. After hospitalization, routine 3.0 T magnetic resonance imaging (MRI) of the parotid area was performed, and 1.5 T MRI or CT scan was used to reconfirm the boundary and scope if there were implants, and the routine preoperative examinations before general anesthesia were improved.

### 2.3 Surgical operation

All the operations were performed by senior attending physicians or deputy chief physicians or above. Based on the site of the tumor, the terminal or trunk method was chosen for the facial nerve dissection; the incision was made by the small S approach of the ear screen; the main trunk of the great auricular nerve was dissected and preserved to avoid postoperative periauricular numbness and discomfort; intraoperative pathological freezing was performed, and after the result was clarified, the glandular stumps were tied by absorptive threads, and if the facial nerve was in direct contact with the skin flap, the tissue patch was used to cover it to avoid Frey's syndrome.

### 2.4 Suture

Plastic and cosmetic buried vertical mattress subcutaneous sutures are used, fully decompensated; a negative pressure drainage tube is routinely left and removed after 3 days; a mandibular elastic sleeve is routinely worn for about 3 weeks after the removal of the drainage tube; patients are instructed to consume a light diet for 2 weeks after the operation; beginning 1 week after removal of the stitches, anti‐scarring medication is applied externally for 6 months.

### 2.5 Statistical analysis

All data were analyzed by SPSS 27.0 software. Data for categorical variables were expressed as the number (frequency), and the chi‐square test was used to compare differences between groups. Data on continuous type variables were subjected to the Shapiro–Wilk test, and *p* > 0.05 indicated that the data were normally distributed. The normally distributed data were expressed as mean ± standard deviation (SD), and the *t*‐test was used to compare differences between groups. *p* < 0.05 was considered statistically significant.

## 3 RESULTS

In the present study, patients with benign parotid tumors consisted of 21 males and 31 females with a male‐to‐female ratio of 1:1.48. The age of the patients was 43.81 ± 13.59 years, ranging from 18 to 71 years, with the largest percentage (50%) in 30–50 years (Table 1). The mean age of males was 43.33 years and that of females was 44.13.

**TABLE 1:** General characteristics.

Characteristics	*N* (%)/mean ± SD (Min–max)
Gender	52 (100%)
Male	21 (40.4%)
Female	31 (59.6%)
Age	43.81 ± 13.59 (18 ~ 71)
<30 years	8 (15.4%)
30 ~ 50 years	26 (50.0%)
>50 years	18 (34.6%)
Tumor size	2.95 ± 0.71 (1.9 ~ 5.5)
≤3 cm	33 (63.5%)
>3 cm	19 (36.5%)
Tumor position	52 (100%)
Left	22 (42.3%)
Right	30 (57.7%)
Pathological type	52 (100%)
Pleomorphic adenoma	31 (59.6%)
Warthin's tumor	13 (25.0%)
Basal cell adenoma	2 (3.8%)
Myoepithelioma	5 (9.6%)
Cystadenoma	1 (1.9%)

**FIGURE 1:**
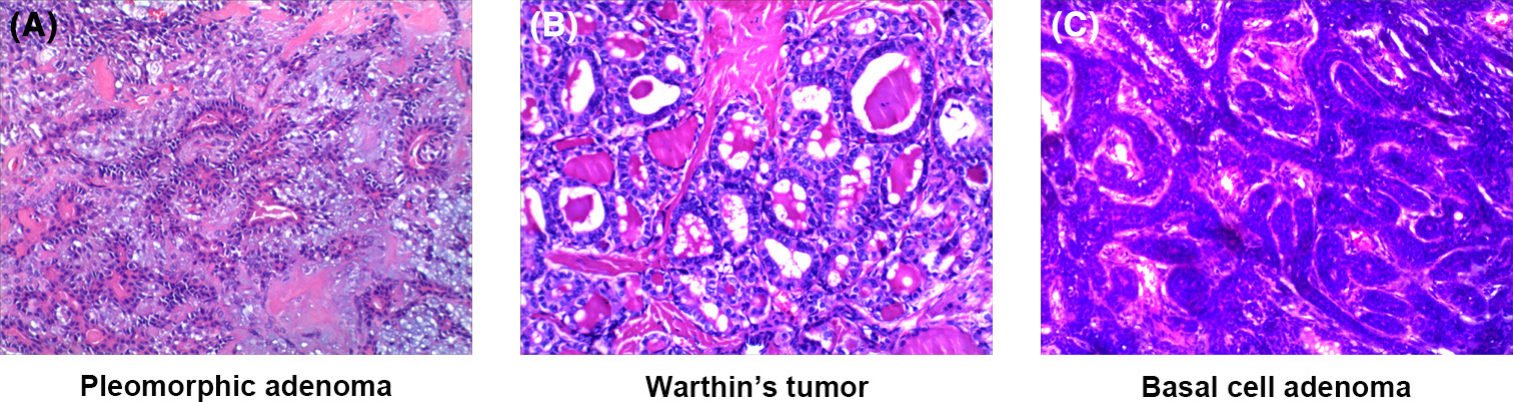
Representative hematoxylin–eosin‐stained images of benign tumors of the parotid gland. (A) Pleomorphic adenoma. (B) Warthin's tumor. (C) Basal cell adenoma.

Histological examination showed that the diameter of benign parotid tumors was 2.95 ± 0.71 cm, with the smallest tumor measuring 1.9 cm in diameter and the largest 5.5 cm. The majority of tumors (63.5%) were less than 3 cm in diameter. Of the 52 cases, 22 tumors (42.3%) were located on the left side of the face and 30 (57.7%) on the right side. Pathologic diagnosis showed that pleomorphic adenomas were the most common (59.6%), followed by Warthin's tumor (25.0%), which together accounted for 84.6% of all cases, with the remaining myoepithelioma (9.6%), basal cell adenoma (3.8%), and cystadenoma (1.9%) being less common (Table 1). A representative hematoxylin–eosin‐stained image of a parotid tumor is shown in Figure 1.

The patients' surgery‐related indicators were as follows: suture time was 14.83 ± 1.61 min, with a minimum of 8 min and a maximum of 18 min; wound healing time averaged 6 days; operative time was 58.90 ± 15.76 min; hospital stay ranged from 4 to 7 days, with a mean hospital stay of 5 days; intraoperative bleeding volume was 38.54 ± 9.15 mL; and three‐day drainage volume was 54.08 ± 13.08 mL (Table 2).

**TABLE 2:** Surgical indicators.

Surgical indicators	Mean ± SD	Min–max
Suture time (min)	14.83 ± 1.61	8–18
Wound healing time (days)	6.33 ± 0.90	5–10
Surgical time (min)	58.90 ± 15.76	25–102
Hospital day (days)	5.12 ± 0.96	4–7
Bleeding volume (mL)	38.54 ± 9.15	25–59
Drainage volume (mL)	54.08 ± 13.08	30–90

Postoperative complications were summarized, and it was found that there were six cases of temporary facial paralysis, one case of salivary fistula, two cases of Frey's syndrome, none of infection, and none of permanent facial nerve manifestations. Sensory numbness in the incision area was observed in six cases within 1 month after surgery, and permanent periauricular numbness was observed in one case after 3 months (Table 3).

**TABLE 3:** Postoperative observation indicators.

Postoperative indicators	*N* (%)/mean ± SD (Min ~ max)
Complications	16 (30.8%)
Salivary fistula	1 (1.9%)
Frey's syndrome	2 (3.8%)
Facial paralysis (temporary)	6 (11.5%)
Numbness of sensation in the incision area (temporary)	6 (11.5%)
Periauricular numbness (permanent)	1 (1.9%)
Sunnybrook score	88.40 ± 10.53 (48 ~ 100)
<80	7 (13.5%)
≥80	45 (86.5%)
VSS score	1.15 ± 0.36 (1 ~ 2)
POSAS score	1.33 ± 0.47 (1 ~ 2)

**FIGURE 2:**
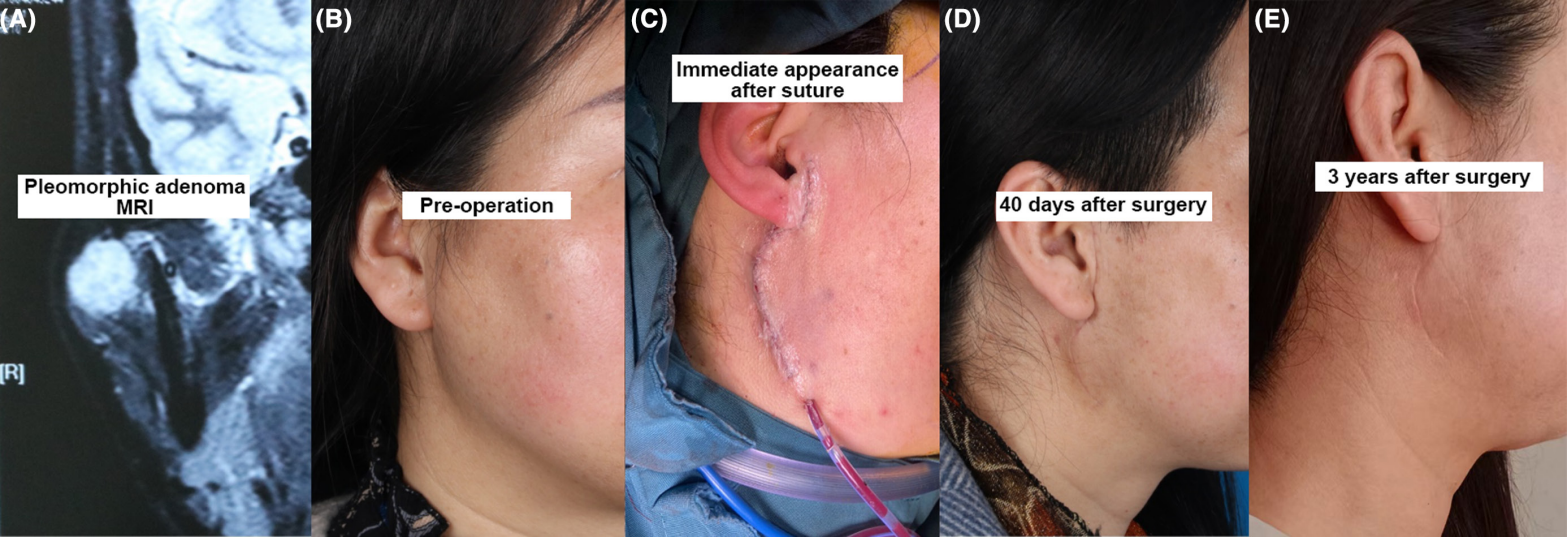
Preoperative and postoperative facial appearance of benign parotid tumors. (A) MRI image of pleomorphic adenoma. (B) Image of the patient's parotid region before surgery. (C) Immediate appearance after parotidectomy sutures. (D) Image of the parotid region 40 days after surgery. (E) Image of the parotid region 3 years after surgery.

At 6 months after surgery, the Sunnybrook facial nerve function score was 88.40 ± 10.53, and the majority (86.5%) of the patients had a Sunnybrook score ≥80, indicating good facial nerve function. The VSS score and the POSAS score were both between one and two, indicating a good scar condition, close to that of normal skin (Table 3). As shown in Figure 2, this is the appearance of the parotid region in a patient with a benign parotid tumor, including preoperatively, after surgical suturing, 40 days after surgery and 3 years after surgery. At 40 days after surgery, the patient's facial scar was more visible, and at 3 years postoperatively, the scar had almost disappeared.

Based on the tumor size, 52 patients were divided into two groups, and statistical analysis revealed no statistically significant differences in the complication rate, Sunnybrook Scale, VSS score and POSAS score between the two groups (*p* > 0.05) (Table 4).

**TABLE 4:** Comparison of postoperative indices according to tumor size.

Tumor size	Complications	Sunnybrook score	VSS score	POSAS score
≤3 cm	8 (24.2%)	86.70 ± 11.77	1.15 ± 0.36	1.30 ± 0.47
>3 cm	8 (42.1%)	91.37 ± 7.30	1.16 ± 0.38	1.37 ± 0.50
*χ* ^2^/*t*	1.065	1.563	0.06	0.476
*p*	0.302	0.124	0.952	0.636

## 4 DISCUSSION

In this study, a total of 52 patients were diagnosed with benign parotid tumors by preoperative MRI, and the most common histologic types were pleomorphic adenomas and Warthin's tumors (59.6% and 25.0%, respectively). Qi et al. found that MRI has excellent diagnostic performance in differentiating benign and malignant tumors of the parotid gland, especially in pleomorphic adenoma, and Warthin's tumor.^6^ Preoperative ultrasound combined with MRI is routinely used to clarify the extent and nature of the tumor, which facilitates the planning of preoperative protocols. The incidence of parotid tumors among maxillofacial salivary gland tumors is reported to be about 80%, with pleomorphic adenomas and Warthin tumors being the most common,^7^ which is consistent with our results. With economic development, patients' demands have gradually increased, requiring shorter hospitalization cycles and recovery times, invisible trauma, and minimal or no postoperative complications. In our group, a total of 52 patients with benign parotid tumors underwent partial parotidectomy with a conventional short S‐incision combined with plastic and cosmetic suturing techniques. The postoperative complication rate of the patients was 30.8%, with no case of facial scarring, one case of salivary fistula, six cases of temporary facial paralysis and two cases of Frey's syndrome. The complication rate of total parotidectomy was lower than that reported by Marie.^8^

Iwai et al. found that different surgical resection modalities were associated with the occurrence of postoperative salivary fistula.^9^ However, Zou et al. found no statistically significant difference in the incidence of postoperative salivary fistula in patients with either partial or complete parotidectomy.^10^ Daniel et al. demonstrated that postoperative compression and avoidance of salivary stimulating foods were significant in reducing postoperative salivary fistula.^11^, ^12^ In our group of 52 patients who routinely wore a mandibular elastic sleeve for 3 weeks or more after removing the drain on the third postoperative day, only one case developed a salivary fistula, mainly because the patient was discharged from the hospital after eating acidic foods and not wearing the elastic sleeve. Six of our cases presented with temporary postoperative facial nerve symptoms, four of which were more closely related to the common trunk of the facial nerve and may have suffered blunt contusion during dissection. Two of the cases had a long history, one of 15 years and one of 20 years, and intraoperatively, the tumor was found to compress the nerve more finely, and there was a distraction contusion in the dissection. In addition, two patients presented with Frey's syndrome. After resection of the tumor, direct contact between the facial nerve and the flap existed in these two patients requiring tissue patch coverage, but the patients refused to implant an allogeneic tissue patch, and indicated that they were comfortable with the complication. Brown protected the auricular and posterior branches of the great auricular nerve intraoperatively for the first time and demonstrated that not only was the incidence of periauricular numbness significantly lower in those with the auricular and posterior branches preserved, but the recovery time was also relatively shorter postoperatively.^13^ In this study, one case showed permanent postoperative sensory numbness, mainly because the postauricular branch was found to be more closely adherent to the tumor, so it was directly resected during the operation to avoid postoperative recurrence.

To avoid postoperative scarring, many scholars have modified the incision for benign tumors in the parotid region, which is mainly divided into preauricular, retroarticular, and preauricular‐postauricular combined incisions, etc. The modified incision can guarantee a good surgical field of vision while reducing the incidence of postoperative complications.^14^, ^15^ In this study, a short S‐incision was used to fully expose the tumor, combined with a modified buried vertical mattress suture and later with topical anti‐scar medication for 6 months. The results showed that most of the patients had good facial nerve function at 6 months postoperatively, with very low POSAS scores, and none of the facial incision areas showed significant scarring. Due to the exposure of the surgical field and the thorough resection of the tumor, there were no cases of recurrence in the 3‐year follow‐up.

This study preliminarily analyzed the effect of modified buried vertical mattress sutures combined with external application of anti‐scarring drugs, which provides a new direction, and valuable reference for the treatment of benign parotid tumors. However, there are some limitations to this study. Due to the small sample size of this study, as well as the fact that other influencing factors, such as the surgeon's experience, operation details and patient compliance, were not included, the findings are somewhat biased. Expanding the sample size and conducting a randomized controlled trial were considered at a later stage to more fully assess the value of its clinical application.

In conclusion, this study highlights the potential of the modified buried vertical mattress suture technique with anti‐scar principles in parotid benign tumor surgery. It shows promise in minimizing scarring and enhancing patient satisfaction, though further validation is needed.

## AUTHOR CONTRIBUTIONS

Ming Zhang contributed to the study conception and design. Xueyuan Yu contributed to the Material preparation and Data collection. Yuan Guo and Maoguo Shu contributed to Data analysis and Software. The first draft of the manuscript was written by Xiangyu Liu. All authors commented on previous versions of the manuscript, read and approved the final manuscript.

## FUNDING INFORMATION

The authors declare that no funds, grants, or other support were received during the preparation of this manuscript.

## CONFLICT OF INTEREST STATEMENT

The authors have no relevant financial or non‐financial interests to disclose.

## ETHICS STATEMENT

This study was approved by the ethics committee of The First Affiliated Hospital of Xi'an Jiaotong University. For research in humans, the research was conducted per the principles of the Declaration of Helsinki. This patient has signed a written informed consent for the use of anonymized images for publication.

## Data Availability

All data generated or analyzed during this study are included in this published article.
